# Fracture Resistance of Roots after Application of Different Sealers

**DOI:** 10.22037/iej.2017.10

**Published:** 2017

**Authors:** Fatemeh Dibaji, Farzaneh Afkhami, Babak Bidkhori, Mohammad Javad Kharazifard

**Affiliations:** a *Department of Endodontics, Dental School, Tehran University of Medical Sciences, International Campus, Tehran, Iran; *; b * General Dentist, Tehran, Iran; *; c * Statistical Consultant, Dental Research Center, Tehran University of Medical Sciences, Tehran, Iran*

**Keywords:** AH-Plus, Bioceramic, Epiphany, Fracture Resistance, iRoot, Resilon, Sealer

## Abstract

**Introduction:**

Vertical root fracture inevitably leads to tooth extraction. Thus, root filling with obturating materials and sealers that can reinforce the tooth would be an ideal way to reduce fracture in root treated teeth. This study aimed to assess the fracture resistance of roots following the application of different sealers including Epiphany, iRoot sealer and AH-plus.

**Methods and Materials:**

Fifty extracted human single-canal premolars without caries, curvature or cracks were used in this study. Tooth crowns were cut to yield 13-mm-long roots. Five roots were put in the negative control group and were left unprepared. Forty-five canals were prepared using ProTaper rotary files up to F3 and were then randomly divided into three groups based on the sealer type (*n*=15). The root canals were filled using cold lateral condensation technique with gutta-percha and AH-Plus sealer, gutta-percha and iRoot sealer and Resilon and Epiphany sealer, in groups one to three, respectively. The roots were then mounted in acrylic molds for fracture resistance testing and subjected to compressive load at a crosshead speed of 1mm/min until fracture. Data were analyzed using the one-way ANOVA.

**Results:**

The mean fracture resistance was 673.38±170.42 N in AH-Plus, 562.00±184.68 N in iRoot, 708.03±228.05 N in Resilon and 592.59±117.29 N in the control group. No statistically significant difference was found between the experimental groups and the negative control group (*P*=0.26).

**Conclusion:**

Application of AH-Plus, bioceramic and Resilon sealers did not change the fracture resistance of roots compared to that of unprepared root canals.

## Introduction

At present, gutta-percha along with sealer is the gold standard of root canal filling [1]. Sealers are capable of filling the voids between gutta-percha cones and the gaps between gutta-percha and dentinal canal walls [2]. Sealing of apical and lateral gaps in the root canal system and adaptation to the dentinal canal walls are the favorable characteristics of ideal sealers [3]. On the other hand, thinning and weakening of root canal walls may occur due to excessive pressure during root canal cleaning and shaping, over-instrumentation, removal of intracanal post, previous endodontic treatment, internal root resorption or dehydration due to the application of irrigating solutions. As a result, the resistance of root canals to functional loads may decrease and the roots become more susceptible to fracture. Therefore, standard principles must be thoroughly followed when filling the root canals [4, 5].

It is believed that root canal sealers that are capable of bonding to root dentin can increase the fracture resistance of endodontically treated teeth [6]. However, studies have yielded controversial results in this regard. A previous study showed no significant difference in fracture resistance of root dentin between root canals filled with Epiphany (which bonds to root dentin) and epoxy resin-based AH-Plus sealer [7]. In contrast, another study showed lower fracture resistance of root dentin in root canals filled with AH-Plus compared to those filled with Epiphany sealer [8].

Bioceramic sealers like iRoot (Innovative BioCeramix Inc, Vancouver, BC, Canada), are available in the form of ready-to-use premixed injectable paste. They are composed of calcium phosphate, calcium silicate, calcium hydroxide, zirconium oxide, fillers and thickening agents [9]. They are capable of forming hydroxyapatite crystals during setting and consequently, chemically bond to root dentin [9, 10].

A previous study demonstrated that application of bioceramic sealer along with ActiVGP cones (Brasseler USA, Savannah, GA, USA) yielded root fracture resistance similar to that of a sound tooth [11]. Also, the role of bioceramic sealers in increasing the fracture resistance of root is reported to be similar to that of AH-Plus, MTA Fillapex and sound tooth [12]. Resilon/Epiphany (Pentron Clinical Technologies, Wallingford, CT) system bonds to dentin and forms a mono-block [13]. Increased fracture resistance of root dentin following the application of this system has been reported in several studies [8, 14]. However, the efficacy of Epiphany sealer has not been compared to that of bioceramic sealers. 

The aim of this *in vitro* study was to assess the fracture resistance of root dentin following the application of iRoot bioceramic sealer, Resilon/Epiphany system and AH-Plus sealer.

## Materials and Methods

***Preparation of specimens:***

Fifty human single-rooted premolars that were recently extracted due to periodontal or orthodontic reasons were selected. Presence of a single canal and absence of root canal curvature and caries were ensured radiographically. Soft tissue residues and calculus were removed using a piezo scaler. After cleaning, the teeth were thoroughly examined with a stereomicroscope (SM X800, Nikon Co, NY, USA) under 20× magnification to ensure absence of cracks or open apices. The teeth were then immersed in 0.5% chloramine-T solution for 7 days. Tooth crowns were then cut using a fissure diamond bur (Teeskavan Co, Tehran, Iran) under copious water irrigation to yield 13-mm-long roots. Of the specimens, 5 were randomly selected as the negative controls. The root canals in the negative control group were not instrumented or filled. The working length of the remaining 45 teeth was determined using a #15 K-file (Mani, Tochigi, Japan). The file was introduced into the canal until the tip was visible at the apex 1 mm was subtracted from this length to yield the working length. The root canals were prepared by ProTaper instruments (Dentsply Maillefer, Ballaigues, Switzerland) using the single length technique up to F3. Also, the root canals were standardized in terms of mesiodistal and buccolingual widths. In between each filing, canals were rinsed with 3 mL of 2.5% NaOCl. After completion of instrumentation, the root canals were rinsed with 2mL of 5.25% NaOCl for 60 sec followed by 1 mL of 17% ethylenediaminetetraacetic acid (EDTA, CinaBartar Co, Tehran, Iran) for 60 sec to remove the smear layer. Final rinse was done with 5 mL of distilled water. The canals were then dried with paper points.

After instrumentation, prepared root canals were randomly divided into three groups of 15 and were filled using cold lateral condensation technique as follows: group one, root canals were filled with gutta-percha (Gapadent Co, Tianjin, China) and AH-Plus sealer (Densply, DeTrey, Konstanz, Germany) which was prepared according to the manufacturer’s instructions. The mixture was applied to canal walls by a master gutta-percha cone (Gapadent Co, Tianjin, China) and the lateral cones were also dipped in sealer and placed in the canals; in group two, canals were filled with gutta-percha and iRoot (Innovative BioCeramix Inc, Vancouver, BC, Canada). According to the manufacturer’s instructions, sealer was injected into the canal to fill the apical one-third. Placement of sealer in the apical third was ensured by taking radiographies. Root canal was then filled with gutta-percha as described above. In group three, canals were filled with Resilon and Epiphany sealer (Epiphany; Pentron Clinical Technologies, Wallingford, CT, USA) as instructed by the manufacturer. First, monomer was applied to the root canals by a paper point and then the canals were filled with Resilon and Epiphany sealer. After cutting the Resilon in this group, the coronal portion of the filling was light cured for 40 sec to achieve an immediate coronal seal.

To allow complete setting of sealers in all experimental groups, the teeth were stored in 100% moisture at 37^°^C for 7 days.

***Fracture resistance testing:***

The roots were vertically mounted in copper molds filled with autopolymerizing acrylic resin (Marlic Medical Industries Co., Eshtehard, Iran) in separate manner. Resin cylinders 10 mm in height and 20 mm in diameter were fabricated as such; 7 mm of the root length was embedded in acrylic resin and 6 mm of it was out of the acryl (13). For fracture resistance testing, roots mounted in acrylic blocks were fixed on the jig of universal testing machine (Z050, Zwick/Roell, Ulm, Germany). Compressive load was applied to the canal orifice by a ball with 4 mm diameter at a crosshead speed of 1 mm/min until fracture. The load at fracture was recorded in Newton (N). 

SPSS software (SPSS 22.0; SPSS, Chicago, IL, USA) was used for data analysis. Data were analyzed using the one-way ANOVA and the level of significance was set at 0.05.

## Results

The mean fracture resistance of the test and control groups is shown in Table 1. One sample Kolmogorov-Simonov test confirmed the normal distribution of data in all groups (*P*<0.05). One-way ANOVA revealed no significant difference in fracture resistance among the experimental and control groups (*P*=0.26). 

## Discussion

Excessive removal of tooth structure during preparation (over-instrumentation) and too much pressure during root canal filling decrease the fracture resistance of endodontically treated teeth [15]. On the other hand, use of irrigants cause dentin dehydration, and decreases its elastic modulus and flexural strength and makes the root more susceptible to fracture [16]. 

Root canal filling materials and sealers are among the potentially recognized factors to increase the fracture resistance of teeth. Thus, for root canal obturation, a filling material must be selected that strengthens the compromised tooth structure, which has been subjected to chemical and mechanical preparation [17]. Thus, the current study evaluated the effect of 3 different sealers on fracture strength of roots. This study compared three root canal filling systems in terms of conferring resistance to root dentin. Based on the results, the highest mean fracture resistance of root dentin was seen in Resilon/Epiphany group followed by gutta-percha/AH-Plus, negative control and gutta-percha/iRoot group. However, the values were not significantly different.

In the current study, the root canals were filled using cold lateral condensation technique because this method is widely used in clinical settings. Shashidhar *et al. *[8], compared the fracture strength of teeth filled with lateral or vertical condensation techniques. The maximum fracture resistance of teeth was obtained after filling the root canals using the lateral condensation technique. Also, several studies have used this technique for root canal filling; thus, our results can be easily compared with those of previous studies [18]. 

It is difficult to standardize the human teeth for assessment of fracture strength because anatomical variations, age and time of extraction of teeth can affect the results [19, 20]. When extracted teeth are used for testing, factors such as mesiodistal and buccolingual width and length of root canals must be standardized [7]. In our study, all roots were standardized in terms of size of preparation, root width and length. It has been stated that preparing the root canals with a round cross-section results in equal distribution of stress in root during filling, and risk of root fracture decreases [21]. For this purpose, rotary files were used for root canal preparation. 

Universal testing machine has been used for measurement of fracture resistance of teeth in many studies [22, 23]. In our study, load was vertically applied along the longitudinal axis of the teeth; because in this method, load entirely transfers to the root [22, 23].

The smear layer is comprised of non-organic particles of calcified tissue along with the necrotic pulp tissue, bacteria and blood cells [24]. It covers the dentinal tubules, prevents the penetration of sealers into these areas and consequently, the sealers cannot confer resistance to root dentin. Thus, the smear layer must be preferably eliminated [25]. In the current study, we used EDTA to remove the smear layer. Due to low surface tension, EDTA easily penetrates into the dentinal tubules and dissolves the smear layer as deep as 2.5 to 4 μm [26, 27]. Thus, the bond and adaptation of sealer to root canal walls increase [27, 28]. Eventually, distilled water was used to neutralize and wash out the remaining irrigating solutions. 

In the current study, iRoot sealer was also used, which is a recently introduced sealer with a calcium silicate base. This sealer does not require any additional material or mixing and has optimal properties such as osteoconductivity and hydrophilicity; also, it is capable of chemically bonding to root canal dentin [7]. Some studies have shown that chemical bond to root dentin improves the fracture resistance of endodontically treated teeth [13, 29]. Moreover, bioceramic sealer is a hydrophilic material with low contact angle that allows for easy flow of the sealer on root canal walls and results in good adaptation and optimal seal *via* mechanical interlocking. On the other hand, very fine, premixed particles of this sealer are injected by a very thin needle and flow over the entire length of the canal [10]. Furthermore, zirconium oxide has been used in the formulation of this sealer, which has high fracture strength, high tensile strength and low Young’s modulus [30].

**Table 1:** The mean (SD), maximum and minimum values of fracture resistance of teeth

**Sealer**	**Mean (SD)**	**Minimum**	**Maximum**
**AH-Plus**	673.38 (170.42)	319.80	876
**iRoot**	562.00 (184.68)	324.94	921.57
**Resilon**	708.03 (228.05)	346.06	1203.56
**Negative control**	592.59 (117.29)	395.62	689.03

Sagsen *et al.* [12] compared the fracture resistance of the root-filled teeth with Resilon/Epiphany sealer, Gutta-percha/AH-26, or gutta-percha/MCS Canal Sealer and reported similar results. They found no significant difference between the two groups. Comparable to our findings, Topçuoğlu *et al. *[31] reported no significant difference in root dentin fracture resistance of root canals filled with gutta-percha/AH-Plus or gutta-percha/Endosequence BC sealer which is another bioceramic sealer.

Epiphany is a dual cure resin-based sealer. Studies have discussed that improved root strength following application of Resilon/Epiphany system is due to the capability of Epiphany sealer in bonding Resilon to root canal dentin and forming a mono-block [8]. Also, Kazandag *et al.* [32] reported that the fracture resistance of AH-26/gutta-percha group was equal to that of Resilon/Epiphany group.

However, in contrast to our findings, Kumar *et al.* [33] found that forces at fracture was significantly higher in Resilon points/RealSeal dual cure sealer than the gutta-percha/AH-plus group. Similarly, Teixeira *et al.* [13] stated that teeth filled with Resilon/Epiphany system had higher fracture resistance than teeth filled with gutta-percha and AH-26 sealer. Such a difference between their results and ours may be due to difference in methodologies. In the study by Ashraf* et al.* [34] sound roots showed higher fracture resistance than roots filled with Resilon/Epiphany and gutta-percha and AH-26 sealer. However, the difference between Resilon filled and sound roots was not significant; this finding was in line with our result. However, higher fracture resistance of roots in Epiphany group compared to AH-26 sealer is in accordance with the findings of Teixeira *et al.* [13], Hammad *et al.* [14], Monteiro *et al.* [35] and Ahlberg *et al.* [36], but different from our findings.

In another study, canals were filled with 3 different systems: glass ionomer-based sealer and cone (ActiV GP obturation system), or two bioceramic sealers (EndoSequence BC or Smartpaste bio obturation systems) and cone. The results showed that all systems increased the fracture resistance of instrumented roots but no significant differences were found between these experimental groups [37].

Search of the literature yielded no study comparing iRoot sealer and Resilon/Epiphany on fracture strength of roots. Since the manufacturers of both sealers claim that they bond to dentin and increase the fracture strength of roots, this study compared these two sealers and revealed that both sealers increased the fracture strength of endodontically treated teeth to the level of sound teeth and had no significant difference in this regard with each other.

## Conclusion

Application of AH-Plus, iRoot and Resilon/Epiphany sealers did not change the fracture resistance of roots compared to that of unprepared root canals.
